# Birth season and environmental influences on blood leucocyte and lymphocyte subpopulations in rural Gambian infants

**DOI:** 10.1186/1471-2172-9-18

**Published:** 2008-05-07

**Authors:** Andrew C Collinson, Pa Tamba Ngom, Sophie E Moore, Gareth Morgan, Andrew M Prentice

**Affiliations:** 1 Directorate of Child and Women's Health, Bramble Ward, Royal Devon and Exeter Hospital (Wonford), Exeter, UK; 2 Nutrition Programme, MRC Laboratories, Banjul, The Gambia; 3 Developmental Medicine (Paediatrics/Immunology), University of Wales, Swansea, UK; 4 MRC International Nutrition Group, London School of Hygiene & Tropical Medicine, London, UK

## Abstract

**Background:**

In rural Gambia, birth season predicts infection-related adult mortality, providing evidence that seasonal factors in early life may programme immune development. This study tested whether lymphocyte subpopulations assessed by automated full blood count and flow cytometry in cord blood and at 8, 16 and 52 weeks in rural Gambian infants (N = 138) are affected by birth season (DRY = Jan-Jun, harvest season, few infections; WET = Jul-Dec, hungry season, many infections), birth size or micronutrient status.

**Results:**

Geometric mean cord and postnatal counts were higher in births occurring in the WET season with both season of birth and season of sampling effects. Absolute CD3+, CD8+, and CD56+ counts, were higher in WET season births, but absolute CD4+ counts were unaffected and percentage CD4+ counts were therefore lower. CD19+ counts showed no association with birth season but were associated with concurrent plasma zinc status. There were no other associations between subpopulation counts and micronutrient or anthropometric status.

**Conclusion:**

These results demonstrate a seasonal influence on cell counts with a disproportionate effect on CD8+ and CD56+ relative to CD4+ cells. This seasonal difference was seen in cord blood (indicating an effect *in utero*) and subsequent samples, and is not explained by nutritional status. These findings are consistent with the hypothesis than an early environmental exposure can programme human immune development.

## Background

Using demographic data from West Kiang in rural Gambia, collected since 1949, we have previously demonstrated a profound birth-season bias in adult deaths, with a large excess of early adult deaths amongst individuals born in July-December [1]. This period includes the annual rains and 'hungry season' arising from depletion of the previous year's food stocks. In combination with a period of intensive agricultural labour, this results in an acute negative energy balance lasting several months in all adults, including pregnant women [2]. The predominance of infectious or infection-related deaths in the historical cohort [1], suggests the programming of immune function by a seasonal component of the fetal or early postnatal environment. Candidate programming factors include seasonal differences in fetal nutrient deprivation, exposure to toxins (e.g. aflatoxin or pesticides), or antigen exposure. These could act directly, or indirectly via priming or suppressive effects of maternal immunological or endocrine signals [2].

The thymus is a potential programming target that is central to the development of adaptive immunity, contributing to long-term maintenance of T-lymphocyte populations [3]. In animal models, maternal under-nutrition has a disproportionately severe impact on thymic growth [4], and there is some evidence of the same phenomenon in humans [5]. Limited evidence suggests a positive association between thymic volume and circulating naïve phenotype CD4+ T-cell numbers [6], and between fetal growth restriction and reduced T-lymphocyte subpopulation counts at birth [7,8], although the latter findings have not been replicated using modern flow cytometric techniques (G. Morgan, unpublished observations). In this Gambian population we have recently described seasonal variations in the proportion of T-cells of recent thymic origin as assessed by T-cell rearrangement excision circles (TRECs) [9]. Studies in other West African children have described seasonality in a variety of immunological measures, including seasonal effects on lymphocyte and T-cell subpopulation counts [10].

This study was designed to test the hypothesis that absolute and percentage lymphocyte subpopulation counts in this community are affected by birth-season with a greater effect on T-cell subpopulations and a maximal discrepancy between January-June (DRY) and July-December (WET) births. Here we describe changes in leucocyte, lymphocyte and CD3+, CD4+, CD8+, CD19+, CD56+ subpopulation counts in 138 rural Gambian infants.

## Results

There were three deaths during follow-up: one unexpectedly at home aged 7 weeks, cause unknown, a second from malaria complicated by severe anaemia aged 34 weeks, and a third from dysentery with septicaemia aged 36 weeks. Two infants left the study during follow-up. Overall, lymphocyte subpopulation data were obtained from 453 (83%) of 545 possible samples.

Mean (range) birth-weight and gestation were 2855 (2020–3900) grams and 38.6 (35.4–41.2) weeks. Birth-weight was similar in the two seasons (p = 0.75, adjusted for gender, gestation and parity), and this remained the case after adjusting for maternal weight. All infants were breast fed from birth, reported introduction of complementary feeds ranging from 8 to 32 weeks. Weight-for-age improved from a median z-score of -0.8 at birth to 0 at 8 weeks, but deteriorated progressively thereafter to -1.9 by 11 months. Seven blood films were positive for malaria parasites out of all the 8, 16 and 52 week visits when the infants were venesected and all samples examined for malaria parasites. There were an additional 28 positive blood films from the remaining four-weekly visits (1309 visits in total), when capillary blood was examined only in febrile infants.

### Effects of birth season

**Figure 1:**
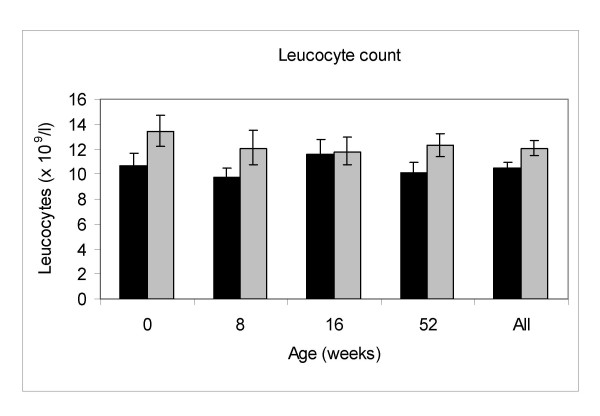
**Leucocyte counts by season of birth (geometric mean and 95% confidence intervals).** Solid black bars represent DRY season births, solid grey bars represent WET season births.

**Figure 2:**
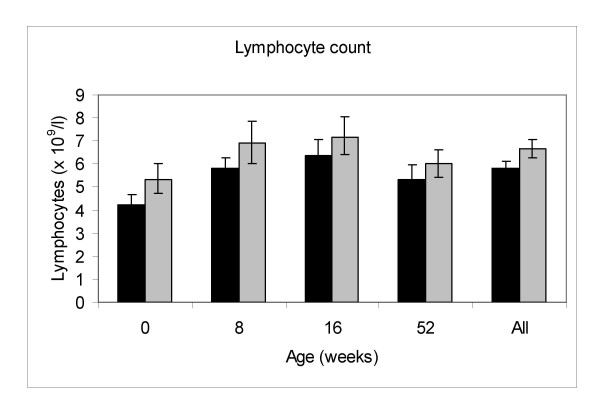
**Lymphocyte counts by season of birth (geometric mean and 95% confidence intervals).** Solid black bars represent DRY season births, solid grey bars represent WET season births.

There was a strong effect of birth season on the total lymphocyte and leucocyte counts at all ages, including at birth (see Figures 1 and 2). The geometric mean cord blood lymphocyte and leucocyte counts were both higher in WET season births compared to DRY season births (p = 0.007 and p = 0.0014 for lymphocyte and leucocyte counts respectively). The postnatal lymphocyte and leucocyte counts were also higher in WET season births (p = 0.0031 and p = 0.0002 for lymphocyte and leucocyte counts respectively, for all ages combined). Overall absolute CD3+, CD8+, and CD56+ counts were positively associated with WET season birth (p = 0.01, 0.0007 and 0.0014 respectively; Table 1). Changes in the absolute lymphocyte count and subpopulations were not independent of a general effect on total leucocyte numbers as no significant association was seen between percentage lymphocyte count (expressed as a percentage of total leucocyte count) and either season of birth or season of sampling. Overall percentage CD4+ lymphocyte counts were negatively associated with wet season birth (p = 0.0017; Table 1), whilst absolute CD4+ counts showed no association with birth season. CD19+ counts showed no significant association with birth season, whether expressed as absolute counts or percentages.

**Table 1:** Geometric mean absolute and percentage lymphocyte subpopulation counts at each age and overall, according to season of birth (values adjusted for gender, gestation, birth weight and monoclonal antibody source; overall values also adjusted for age at measurement).

		**Absolute counts (× 10^9^/L)**			**Percentage counts**		
		
	**Week**	**DRY season birth**	**WET season birth**	**p-value**	**DRY season birth**	**WET season birth**	**p-value**
**CD3+**	0	2.30	2.53	0.41	51.8	50.5	0.70
	8	3.57	4.35	0.029	58.7	58.2	0.77
	16	4.10	5.26	0.014	57.7	59.0	0.66
	52	3.32	3.72	0.26	60.9	61.9	0.71
	All	3.40	3.85	0.01	57.1	56.2	0.47

**CD4+**	0	1.45	1.73	0.17	35.0	33.8	0.65
	8	2.49	2.73	0.26	40.8	37.0	0.017
	16	2.80	3.28	0.05	39.1	37.4	0.34
	52	2.07	2.19	0.55	38.4	36.7	0.27
	All	2.26	2.41	0.15	37.8	35.1	0.0017

**CD8+**	0	1.29	1.44	0.44	27.2	27.7	0.82
	8	0.98	1.47	0.011	16.3	19.0	0.055
	16	1.34	1.65	0.093	18.7	18.7	0.97
	52	1.12	1.28	0.22	20.9	21.6	0.60
	All	1.16	1.42	0.0007	20.4	21.4	0.18

**CD19+**	0	0.78	0.73	0.68	17.4	14.6	0.084
	8	1.88	1.94	0.79	32.7	30.7	0.37
	16	1.97	2.50	0.034	32.9	34.7	0.39
	52	1.50	1.51	0.95	27.7	25.5	0.32
	All	1.54	1.66	0.23	27.5	25.9	0.12

**CD56+**	0	0.84	0.78	0.68	19.1	16.0	0.14
	8	0.27	0.35	0.095	4.8	5.4	0.34
	16	0.25	0.33	0.028	4.1	4.5	0.38
	52	0.21	0.29	0.0052	3.9	4.7	0.036
	All	0.33	0.42	0.0014	6.1	6.5	0.17

**CD4/CD8 ratio**	0				1.3	1.2	0.70
	8				2.5	1.9	0.018
	16				2.1	2.0	0.69
	52				1.8	1.7	0.32
	All				1.9	1.6	0.013

### Age-related changes

Geometric mean absolute and percentage lymphocyte counts were highest at 16 weeks and significantly lower at 52 weeks (p = 0.0056 and p ≤ 0.0001 respectively). Subpopulation counts at each age are shown in Table 2. Percentage CD4+ counts remained unchanged from birth. A number of age-related changes were observed compared to cord blood. Percentage CD3+ at 52 weeks were 7.8% higher (p < 0.0001) and percentage CD8+ counts were lower at 8, 16, and 52 weeks (p < 0.001), although there was a significant increase between 16 and 52 weeks (p = 0.003). The absolute counts for CD3+ were higher at 8 weeks (p = 0.005) and CD4+ higher at 8, 16 and 52 weeks (p < 0.0001). Absolute and percentage CD19+ counts rose from birth to 16 weeks but declined between 16 and 52 weeks (p < 0.0001 for each comparison). Absolute and percentage CD56+ count fell sharply from birth to 8 weeks (p < 0.0001 for both comparisons) but remained constant thereafter.

**Table 2:** Absolute and percentage lymphocyte surface marker counts.

**Surface marker**	**Age (weeks)**	**Absolute count (× 10^9^/l)**		**Percentage count**	
		
		**n**	**Geometric mean (95% CI)**	**n**	**Geometric mean (95% CI)**
**CD3+**	0	72	2.55 (2.32–2.80)	73	55.1 (52.1–58.3)
	8	114	3.58 (3.28–3.91)	120	57.5 (55.8–59.3)
	16	114	3.75 (3.43–4.11)	117	55.5 (53.2–57.9)
	52	103	3.64 (3.34–3.98)	105	62.9 (60.9–64.9)

**CD4+**	0	78	1.79 (1.62–1.97)	79	38.1 (35.9–40.4)
	8	119	2.40 (2.22–2.59)	125	38.7 (37.2–40.3)
	16	120	2.49 (2.31–2.69)	123	36.9 (35.5–38.3)
	52	103	2.17 (1.98–2.38)	105	37.6 (36.0–39.2)

**CD8+**	0	78	1.24 (1.10–1.40)	79	26.7 (24.9–28.6)
	8	119	1.11 (0.99–1.24)	125	17.6 (16.4–18.9)
	16	120	1.21 (1.08–1.35)	123	18.0 (16.9–19.1)
	52	103	1.22 (1.10–1.37)	105	21.3 (20.0–22.7)

**CD19+**	0	60	0.77 (0.67–0.88)	60	16.0 (14.5–17.6)
	8	96	1.91 (1.72–2.12)	96	31.8 (29.8–33.9)
	16	109	2.22 (2.00–2.47)	109	33.8 (31.8–35.8)
	52	105	1.59 (1.42–1.78)	105	27.5 (25.5–29.7)

**CD56+**	0	62	0.83 (0.71–0.98)	63	17.8 (15.8–20.1)
	8	89	0.30 (0.26–0.35)	95	5.1 (4.6–5.7)
	16	103	0.28 (0.25–0.32)	106	4.3 (3.9–4.7)
	52	103	0.25 (0.23–0.28)	105	4.3 (3.9–4.7)

### Evidence of immunological phenotype

Cord blood percentage CD4+ and CD4+/CD8+ ratios at birth were predictive of levels at 8 weeks (R = 0.3 (p = 0.003), and R = 0.28 (p = 0.0054) respectively). These, in turn and all other subpopulation percentages predicted levels at 16 weeks p ≤ 0.0001 for CD4+, CD8+, CD19+ and CD56+; p = 0.004 for CD3+). The strength of correlation between counts at 8 and 16 weeks ranged from R = 0.26 (CD3+) to R = 0.64 (CD4+).

For percentage CD4+ and CD56+ counts and the CD4+/CD8+ ratio 16 week counts predicted 52 week counts (R = 0.05 (p ≤ 0.0001) for CD4+ and CD56+; R = 0.30 (p = 0.0011) for CD4+/CD8+). The CD4+ and CD19+ 8 week percentage counts at 52 weeks correlated weakly with their respective counts at 8 weeks (R = 0.36, p = 0.004 for CD4+; R = 0.28, p = 0.006 for CD19+).

### Effects of concurrent nutritional status and birth size

We found no association with current weight or weight-for-age standard deviation score for any of the subpopulations at birth, 8, 16 or 52 weeks or overall, whether expressed as absolute or percentage counts. Overall absolute CD19+ counts were positively associated with concurrent plasma zinc concentration (p = 0.0068). There were no other observed associations between any lymphocyte subpopulation and concurrent plasma zinc, retinol or vitamin C concentration.

If birth weight was included in the regression model, absolute CD8+ and CD3+ counts at 8 weeks and absolute CD4+ counts at 52 weeks were positively associated with birth mid-upper-arm circumference (MUAC) (p = 0.0079, 0.0047, and 0.0035 respectively). Overall absolute counts of these subpopulations were also associated with MUAC at birth (p = 0.0011, 0.0014 and 0.0056 for CD3+, CD4+ and CD8+ respectively). A small number of other associations were found with both head circumference and crown-heel length.

### Effects of gender, gestation and birth size

Overall CD4+ and CD3+ counts were negatively associated with gestational age at birth (p = 0.0017 for absolute and p ≤ 0.0001 for percentage CD4+ counts; p = 0.012 for absolute and p = 0.0006 for percentage CD3+ counts). There was no association of the measured populations and subpopulations with gender or length of gestation at any age or overall.

## Discussion

A strong seasonal effect on circulating leucocyte counts was found in cord blood and at every age of measurement in infancy. This was manifested in consistently higher leucocyte counts in the WET season, with a magnitude of 1,600 leucocytes per mm^3 ^in relation to season of birth, or 2,300 leucocytes per mm^3 ^in relation to season of sampling. These changes were accompanied by a corresponding effect on absolute lymphocyte subpopulations counts; this was selective in that it was not seen in the CD4+ subpopulation. The lack of an observed seasonal effect on percentage lymphocyte counts suggests that seasonal factors lead mainly to a non-specific increase in circulating leucocyte counts. Correspondingly, absolute but not percentage CD3+, CD8+, and CD56+ counts were positively associated with WET season birth. Seasonal fluctuations in the burden of infection provide a plausible explanation for the seasonality, since malaria transmission is intensely seasonal in The Gambia [11], and malaria parasitaemia, diarrhoea and levels of the plasma acute phase reactant alpha-1 antichymotrysin (ACT) all showed strong seasonal patterns in this cohort. However, we did not find consistent correlation between lymphocyte subpopulation counts and concurrent plasma ACT, and found no association between any cell population counts and placental malaria status assessed by histological examination (data not shown). Asymptomatic malaria has been shown to be associated with a decreased percentage CD4+ count in 3–6 year old West African children [12]. In the present study however, parasitaemia was detected in only 7 blood samples at 8, 16 and 52 weeks [13], and the overall effect was of a relative increase rather than decrease in lymphocyte and leucocyte numbers in the months of peak malaria transmission.

The observed selective effect on CD8+ and CD56+ cells relative to the CD4+ subpopulation could be explained by seasonal variation in exposure to viral pathogens [14]. Studies in The Gambia have reported seasonal variations in the incidence of clinical presentation with respiratory syncitial virus (RSV) [15] and rotavirus infection [16], with peak rates of clinical infection in December-January

Seasonal fluctuations in leucocyte and lymphocyte subpopulation counts have been reported previously in West African children [10]. In their study, Lisse *et al *found seasonal influences that were strongest in children aged 3 to 5 years, although children aged 0 to 35 months had slightly higher leucocyte counts in the wet season [10]. However, in contrast to the present study, Lisse *et al *reported lower lymphocyte counts in wet season samples. The authors found no seasonal effect on CD4+ cell counts in the younger age group, but they did report an increase in CD8+ counts relative to CD4+ counts in the wet season.

Our results do not permit a reliable distinction of prenatal from postnatal seasonal influences, but it is notable that the effect on total lymphocyte and leucocyte counts was no less pronounced in cord blood. This confirms that a seasonal influence, infective or otherwise, operates in prenatal life. Since mothers in this community are subject to the same seasonality of infectious and parasitic disease exposure, it seems probable that the presence of maternal infection boosts leucocyte and lymphocyte counts in the fetal circulation. It is also likely that the same factors operating at the time of postnatal measurements contributed to the observed seasonality.

We found no effect of birth weight on total leucocyte count, lymphocyte or subpopulation counts. However, the results showed a consistent positive influence of birth MUAC on the overall absolute CD3+, CD4+ and CD8+ counts, after adjusting for birth-weight. For CD3+ and CD8+ this result was also significant in the separate analysis of subpopulation counts at 52 weeks, and for CD4+ counts the effect was also significant at 8 weeks. These results appear robust, since they are consistent in relation to each of the measured T-cell surface markers, and significant in analyses of both the grouped data from all ages of sampling and results from samples at specific ages. These results suggest that at a given birth weight in the range included in this study, infants with a greater MUAC have higher circulating T-cell counts in infancy.

The results provide little evidence that nutritional status in infancy affects circulating leucocyte, lymphocyte and lymphocyte subpopulation counts. The only significant association observed was a strong positive correlation between infant plasma zinc status and absolute CD19+ counts. The interpretation of this finding within the current data is limited, but could support a role for zinc in the early development of B lymphocytes. However, given the almost universally accepted view that malnutrition impairs immune function, the general lack of association between the available measures on nutrient status and leukocyte counts merits some reflection [17]. Numerous previous studies (for example [8,18-20]), reviews (including [21-23]), and leading articles [24], have reported a major impact of malnutrition on the human thymolymphatic system. It is possible that the immunological effects of acute protein-energy malnutrition – the context of many reports of malnutrition-related immunodeficiency – may differ from those of the chronic growth failure seen in this study. A detailed report of factors affecting leucocyte counts and T-lymphocyte subsets in West African children similarly found no clear association of any haematological values with height-for-age or arm circumference, with the exception of higher total leucocyte counts in severely stunted children [10], and a recent study of immune function in a large cohort of rural Gambian children aged 6–9 y found no consistent effects of anthropometric or micronutrient status on delayed-type hypersensitivity reactions or humoral responses to rabies or pneumococcal polysaccharide vaccines [25]. Our observation of substantial within-subject correlation between successive cell subpopulation counts suggests strongly that the measures used in this study were robust and that they described persisting characteristics of immunological phenotype at the individual level. Age-related changes in lymphocyte subpopulations of healthy subjects have been described in cohorts with comparable or smaller numbers of infant subjects than our own [10,26-28], but whereas studies in other populations have reported trends in infancy [27,28], there is little reference data for African infants. Table 3 summarises the subpopulation counts reported by the present study alongside data from three other descriptive infant studies [10,26,29].

**Table 3:** Lymphocyte subpopulation data from the present study and other published studies (^1^present study, ^2 ^[10], ^3 ^[26], ^4 ^[29].

**Subpopulation (absolute counts (× 10^9^/l))**		**Age (months)**	**The Gambia^1^**	**Guinea Bissau^2^**	**UK^3^**	**Singapore^4^**
**Absolute counts**	**CD3+**	0	2.6		3.1	1.7
		2	3.6		2.5	
		12	3.6			2.7
	**CD4+**	0	1.8		1.9	1.4
		2	2.4	2.5		
		4	2.5	2.5		
		12	2.2		2.2	1.8
	**CD8+**	0	1.2		1.5	
		2	1.1	1.2		
		12	1.2		0.9	
	**CD19+**	0	0.8		1	
		12	1.6		0.9	
	**CD56+**	0	0.8		0.9	
		12	0.3		0.5	

**% counts**	**CD3+**	0	55		55	67
		12	62		64	63
	**CD4+**	0	38		35	47
		2	38	38		
		12	37	37	41	37
	**CD8+**	0	26		29	20
		2	17	19		
		12	21		21	19
	**CD19+**	0	16		20	
		12	27		23	
	**CD56+**	0	18		20	
		12	4		11	

**Ratio**	**CD4+/CD8+**	0	1.5	2.3	1.2	
		12	1.8	2.0	1.9	

## Conclusion

In conclusion, against a background of defined nutritional patterns and consistent individual immunological phenotype, we have shown an effect of birth season on circulating leucocyte, lymphocyte and lymphocyte subpopulation counts, with evidence for a seasonal effect *in utero*. The significance of these findings is strengthened by the fact that analyses were carried out in relation to a single *pre hoc *definition of season, selected in the light of previous data relating birth season to adult mortality in this community [1]. We found no association of lymphocyte subpopulation numbers with birth size or cord plasma micronutrient status, but evidence that measures of proportionality (MUAC) at birth may be more sensitive than birth weight as markers of prenatal influences linked to fetal growth.

The relevance of these early seasonal effects on lymphocyte subpopulation counts to the putative programming of long-term immunity remains to be established. Measures of functional immunity in the same cohort [13] and in a separate cohort study of 472 West Kiang children, found no effect of birth season [30]. In a small cohort of healthy young Gambian men from the same population (n = 25), no effect of perinatal nutritional exposures (assessed by season of birth and birth weight) was observed on T lymphocyte kinetics [31]. In a population of Pakistani adults however, lower birth weight was associated with a weaker antibody response to a polysaccharide vaccine, suggesting possible early-life influences on long-term deficits in B-cell mediated immunity [32]. Further work is required to fully understand the long-term implications of early-life seasonal influences on the immune phenotype demonstrated.

## Methods

### Birth cohort

One hundred and thirty-eight infants were recruited antenatally representing 94% of all singleton live-births within five participating West Kiang villages. The climate, economy, and predominant ethnicity of West Kiang are typical of rural Gambia as a whole. Multiple births and infants with birth weight below 1.5 kg, gestation less than 34 completed weeks, or major congenital malformation, were excluded. Of 184 identified pregnancies, 9 (5%) resulted in spontaneous abortion, 24 (13%) were multiple pregnancies or delivered outside the district, and the fates of a further 2 were unknown. Of the remaining 149 infants, 10 mothers (7%) declined to participate and one infant with congenital hydrocephalus was excluded. Seventy-five infants were male. At the time of the present study, HIV prevalence was estimated at 1.7% in reproductive Gambian women [33]. None of the mothers in this study were known to be HIV infected. Breast-feeding remains almost universal in this community, and there are no local sources of commercial formula-feeds.

### Ethics

The study was approved by the Joint Gambian Government/Medical Research Council Ethical Committee. Subjects were recruited with the informed consent of mothers following an explanation of the study in their own language.

### Clinical measurements

Birth-weight and gestational age (by Dubowitz score [34]) were measured by the principal investigator (ACC) in parallel with a trained field assistant within 72 hours of birth (mean 25, range 1–69 hours). Gestation was calculated from the mean of the two independent scores. Anthropometric measures at birth and every four weeks thereafter were recorded using the same regularly validated equipment for all subjects. Measurements were made using standard techniques and expressed as Z-scores relative to the WHO standards. Mothers were weighed lightly clothed and without shoes using portable weighing scales.

Growth and morbidity were assessed fortnightly throughout infancy. Cord blood was collected at birth (10 ml) and venous samples obtained at 8, 16 and 52 week of age (3, 4 and 6 ml respectively). Capillary blood was examined by thick film for malaria parasites every four weeks, in any infant with intercurrent fever of 37.5°C or above, and on each venous blood sample. Plasma alpha-1 antichymotrypsin (ACT) was also measured at 8 weeks and 52 weeks as an indicator of systemic acute-phase inflammatory responses. Infant feeding status was coded at each visit.

### Laboratory Methods

T-lymphocyte (CD3+, CD4+, CD8+, B-cell (CD19+), and natural killer cell (CD56+) subpopulations were enumerated in whole blood samples at birth (cord blood) and at 8, 16 and 52 weeks of age by flow cytometry (FACScalibur, Beckton Dickinson UK Ltd, Oxford, UK) using appropriate isotypic controls (Cyto-Stat, Beckman-Coulter International S.A., Nyon, Switzerland). EDTA anticoagulated whole blood samples were labelled within 6 hours of collection, red cells were lysed according to the manufacturer's instructions, and the labelled cells stabilised and fixed. Stabilised samples were maintained at 4–8 degrees centigrade and analysed within 7 days at the Medical Research Council Laboratories, Fajara, the Gambia.

Location of the lymphocyte gate, and freedom from monocyte contamination, were assessed using monoclonal antibodies to CD14+ and CD45+. The sum of CD4+8+19+56 was calculated as a second internal control, with values between 95% and 105% accepted as confirming acceptable purity [26,29]. Absolute counts of each cell sub-population were calculated in relation to whole blood lymphocyte counts, measured as full blood count and white cell differential on the same fresh blood sample (Celloscope 1260, Analys Instrument AB).

Plasma ACT and micronutrient levels were measured at MRC Human Nutrition Research in Cambridge, UK. Plasma ACT and vitamin C levels assay on a Cobas-Bio centrifugal analyser (F.Hoffman-la-Roche Ltd, Basel, Switzerland). Prior to freezing, plasma samples for vitamin C analysis were mixed with an equal volume of 10% metaphosphoric acid in order to deproteinize the plasma. Plasma zinc concentrations were determined colorimetrically using a commercial kit (Wako Chemicals, Nauss, Germany). Plasma levels of retinol and carotenoids were measured by high-pressure liquid chromatography (HPLC).

### Statistical analysis

All data were analysed using Data Desk Version 6 (Data Description Inc., Ithaca, New York). Population distributions were log-transformed prior to analysis. Effects on cell counts were assessed at each discrete age of sampling, and in datasets of samples at all ages combined, adjusted for days postnatal age. Cell counts were subjected to analysis of variance against defined exposure measures and potential confounders. Chi-squared and t-tests were used where appropriate.

Exposure effects were analysed with and without adjustment for gender and gestation. Birth weight has previously been shown to be affected by birth season in this community [1]. Therefore to assess for independent influences, associations of cell counts with birth weight were corrected for birth season and vice versa. Associations with birth size were analysed adjusting for current size at time of sampling, and effects of birth weight also adjusted for post-partum maternal weight to give a better assessment of deviant fetal growth. Potential associations between cell counts and proportionality at birth were tested by analysing outcomes separately against crown-heel length, head circumference and MUAC at birth, and repeated with adjustment for birth weight. Analyses in relation to head circumference at birth were also analysed after adjusting for crown-heel length.

Outcome associations were considered significant at a level of p < 0.01. Results were further scrutinised for consistency of the association with the *pre-hoc *hypotheses, and in the light of corroborative or contradictory evidence from the rest of the data (for example, the same comparison at other ages).

The outcome analyses at birth and 52-weeks of age were performed twice: first on the complete data sets, and then on subsets of data that excluded cord blood samples below 90% CD45+ cells in the lymphocyte gate (n = 7), and 52-week samples below 92% (n = 8). Descriptive statistics for the birth and 52-week samples were similarly calculated after excluding samples beyond these minimum levels of purity.

## Authors' contributions

ACC, SEM and AMP conceived and initiated the study. ACC conducted all fieldwork and data collection in The Gambia, conducted the statistical analyses and wrote the preliminary draft of the manuscript. PTN was responsible for all flow cytometric analyses. All authors contributed to and approved the final version of the manuscript. None of the authors had a conflict of interest to report.
